# High rates of multidrug resistance among uropathogenic *Escherichia coli* in children and analyses of ESBL producers from Nepal

**DOI:** 10.1186/s13756-016-0168-6

**Published:** 2017-01-11

**Authors:** Narayan Prasad Parajuli, Pooja Maharjan, Hridaya Parajuli, Govardhan Joshi, Deliya Paudel, Sujan Sayami, Puspa Raj Khanal

**Affiliations:** 1 Department of Clinical Laboratory Services, Manmohan Memorial Medical College and Teaching Hospital, P.O.B.: 15201, Swayambhu, Kathmandu, Nepal; 2 Department of Pediatrics, Manmohan Memorial Medical College and Teaching Hospital, Kathmandu, Nepal; 3 Department of Laboratory Medicine, Manmohan Memorial Institute of Health Sciences, Kathmandu, Nepal

**Keywords:** Urinary tract infection, Children, *E coli*, ESBL, Nepal

## Abstract

**Background:**

Emergence of Extended-spectrum beta-lactamase producing *Escherichia coli* causing urinary tract infections (UTI) among pediatric patients is an increasing problem worldwide. However, very little is known about pediatric urinary tract infections and antimicrobial resistance trend from Nepal. This study was conducted to assess the current antibiotic resistance rate and ESBL production among uropathogenic *Escherichia coli* in pediatric patients of a tertiary care teaching hospital of Nepal.

**Methods:**

A total of 5,484 urinary tract specimens from children suspected with UTI attending a teaching hospital of Nepal over a period of one year were processed for the isolation of bacterial pathogens and their antimicrobial susceptibility testing. *Escherichia coli* (*n* = 739), the predominant isolate in pediatric UTI, was further selected for the detection of ESBL-production by phenotypic combination disk diffusion test.

**Results:**

Incidence of urinary tract infection among pediatric patients was found to be 19.68% and *E coli* (68.4%) was leading pathogen involved. Out of 739 *E coli* isolates, 64.9% were multidrug resistant (MDR) and 5% were extensively drug resistant (XDR). Extended spectrum beta lactamase (ESBL) was detected in 288 (38.9%) of the *E coli* isolates.

**Conclusion:**

Alarming rate of drug resistance among pediatric uropathogens and high rate of ESBL-producing *E. coli* was observed. It is extremely necessary to routinely investigate the drug resistance among all isolates and formulate strict antibiotics prescription policy in our country.

## Background

Urinary tract infection (UTI) is among the most common causes of febrile illness in children requiring antimicrobial treatment [1]. Worldwide, an estimated 8% of girls and 2% of boys experience at least one episode of UTI by the age of seven years and recurrence occurs in 12-30% of them within a year [2]. Pediatric UTI in many instances, remain under-diagnosed because of the absence of specific symptoms and signs, particularly in infants and young children [2, 3]. Therefore, accurate diagnosis and appropriate use of antimicrobials for treatment and prevention of urinary tract infections (UTIs) is vital to reduce the burden and also to prevent the possible long-term consequences [4].

*Escherichia coli* have been recognised as the most common pathogen accounting for majority of urinary tract infections in children [5]. Antimicrobial therapy, usually of traditional antibiotics, is commonly prescribed to treat urinary tract infections in pediatric patients. However, increased rates acquired resistance in *E. coli* has made usual antibiotics less acceptable choice for empirical therapy in recent years [1]. The most common mechanism associated with acquired resistance in *E. coli* and other *Enterobacteriaceae* is the production of hydrolytic enzymes called β-lactamases [6, 7]. Extended-spectrum β-lactamase (ESBL), a major beta lactamase enzyme, has the ability to hydrolyze oxyimino-cephalosporins, and monobactams but not cephamycins or carbapenems and inhibited in-vitro by inhibitors such as clavulanic acid, sulbactam and tazobactam [8]. Since their evolution in 1983, more than 300 types of ESBLs have been identified in various members of the family *Enterobacteriaceae* and other non-enteric organisms [3, 6]. The infections associated with these ESBL producing isolates are difficult to treat because of their resistance towards beta lactam agents and also due to the emergence of co-existing resistance determinants such as aminoglycosides and fluoroquinolones [7]. Moreover, emergence of ESBL producing bacteria, particularly *E. coli* and *K. pneumoniae* causing pediatric urinary tract infections is a worldwide concern [9]. Options for the treatment of such multidrug resistant (MDR) gram negative bacterial infections are generally limited, and very few antibiotics are approved for use in children [10].

In Nepal, pediatric UTIs are usually treated empirically because of the unavailability of standard therapeutic guidelines and local susceptibility data [11, 12]. Knowledge of the etiological agent of UTIs and their antimicrobial resistance patterns in our setting may help clinicians in choosing the appropriate antimicrobial treatment. Moreover, most of the studies on pediatric urinary tract infections caused by multidrug resistant and ESBL producing bacteria have been reported from western world [10, 13], but the same from South Asian region including Nepal are scarce on the published literature [14]. In this perspective, the present study was designed to investigate the clinical isolates of multi-drug resistant and ESBL producing *Escherichia coli* causing urinary tract infections in children visiting a tertiary care teaching hospital in Nepal.

## Methods

### Study design and setup

A cross-sectional study was carried out for 1 year (June 2015 - May 2016) in the department of Microbiology and Pediatric Medicine, Manmohan Memorial Medical College and Teaching Hospital (MMCTH), a tertiary care hospital with 500 patient beds in Kathmandu, the capital city of Nepal. Study hospital is a referral center with medical, surgical, gynecological, pediatric, geriatric and other specialties.

### Inclusion and exclusion criteria

During the study period, children up to 14 years of age presented to the pediatric outpatient department or admitted to pediatric inpatient ward with a clinical diagnosis of UTI were included. The clinical diagnosis of UTI was made by respective unit pediatrician in the presence of fever and/or any of the symptoms such painful micturition, increased frequency, burning micturition, or suprapubic pain/flank pain. Those children who had previous known history of antimicrobial therapy within 48 h prior to attending the hospital and samples which grew more than one type of organism was considered as contaminated and hence, excluded from the study.

### Laboratory methods

A total of 5,484 non-repetitive urine specimens (Midstream, Suprapubic, Catheter aspirated and Clean catch) representing urinary tract infections in pediatric patients (0-14 years) were processed semi-quantitatively by inoculating 0.001 ml of the specimen (by using a calibrated wire loop) onto the cystine lactose electrolyte deficient (CLED) agar for the isolation and identification of significant uropathogens [15]. The inoculated plates were incubated for 24 h at 37 °C in aerobic atmosphere. Growth of a single organism with a count of ≥10^5^colony-forming units (CFU)/ml were considered to represent the infection and were identified using appropriate routine identification methods including colony morphology, Gram-stain, and an in-house set of biochemical tests [15]. *Escherichia coli,* the predominant uropathogen, was selected for the determination of antimicrobial susceptibility as well as identification of the multidrug resistant (MDR), extensively drug resistant (XDR) and extended spectrum beta lactamase (ESBL) producing isolates.

### Antimicrobial susceptibility testing

The susceptibility of bacterial isolates against different antibiotics was tested by the disk diffusion method [modified Kirby-Bauer method] on Mueller Hinton agar (Hi-Media, India) following standard procedures recommended by the Clinical and Laboratory Standards Institute (CLSI), Wayne, USA [16]. Antibiotics that were tested in our study include Ampicillin (AMP 25 μg), Amoxycillin clavulanate (AMC20/10 μg), Aztreonam (30 μg) Gentamycin (GEN10μg), Ciprofloxacin (CIP5μg), Levofloxacin (LEV5μg) trimethoprim sulfamethoxazole/cotrimoxazole (COT30μg), Cephalexin (CN30 μg), Cefixime (CFM5μg), Ceftriaxone (CTR30μg), Ceftazidime (CAZ30μg), Piperacillin tazobactam (PIT 100/10 μg), Imipenem (IMP 10 μg), Meropenem (MRP 10 μg) Tigecycline (TGC30μg), and Colistin sulphate (CT10μg) (HiMedia Laboratories, India). Interpretations of antibiotic susceptibility results were made according to the zone size interpretative standards of CLSI [16]. *Escherichia coli* ATCC 25922 was used as a control organism for antibiotic susceptibility testing.

### Identification of Multidrug Resistant (MDR), Extensive Drug Resistant (XDR) and potential ESBL *Escherichia coli*

MDR and XDR isolates were identified according to the combined guidelines of the European Centre for Disease Prevention and Control (ECDC) and the Centers for Disease Control and Prevention (CDC) [17]. In this study, the isolate resistant to at least one antimicrobial from three different group of first line drugs tested was regarded as multidrug resistant (MDR). Extensively drug resistant (XDR) isolates were identified when the isolates are resistant to at least one agent in all but two or fewer antimicrobial categories (i.e., bacterial isolates remain susceptible to only one or two categories).

Isolates of *E. coli* were examined for their susceptibility to third generation cephalosporins by using Ceftazidime (30 μg) and Cefotaxime (30 μg) disks. If the zone of inhibition (ZOI) was ≤25 mm for Ceftriaxone, ≤22 mm for Ceftazidime and/or ≤27 mm for Cefotaxime, the isolate was considered a potential ESBL producer as recommended by CLSI and further tested by confirmatory methods [16].

### Confirmatory test of ESBL

Isolates considered potential ESBL producers by initial screening were emulsified with nutrient broth to adjust the inoculum density equal to that of 0.5 Mac Farland turbidity standards. Combination Disk test (CDT), as recommended by the CLSI, was performed in all isolates presumed to be ESBL producers. In this test, Ceftazidime (30 μg) disks alone and in combination with clavulanic acid (Ceftazidime + clavulanic Acid, 30/10 μg) disks, were applied onto a plate of Mueller Hinton Agar (MHA) which was inoculated with the test strain and then incubated in ambient air for 16-18 h of incubation at 35 ± 2 °C. Isolate that showed increase of ≥ 5 mm in the zone of inhibition of the combination discs in comparison to that of the Ceftazidime disk alone was considered an ESBL producer [16].

### Data analysis

The information regarding patient’s profile and the results were entered into a computer program. Data analysis was carried out using the Statistical Package for Social Sciences [SPSS^TM^] version 20.0 [IBM, Armonk, NY, USA] and presented in percentage base distribution. Data with p value of less than 0.05 (CI-95%) was regarded as significant.

### Ethical consideration

Written approval was taken from Institutional Review Committee of Manmohan Memorial Institute of Health Sciences (MMIHS) after submitting and presenting research proposal. Written informed consent was taken from every patient or their guardians before enrollment into the study.

## Results

### Patient demographics

During the study period, a total of 5,484 representative specimens of urinary tract from pediatric patients suspected with urinary tract infections were processed. Among total clinical specimens, 1079 (19.68%) were found with growth of at least one significant pathogen confirming urinary tract infection (UTI). Female (659, 61.0%) were most affected group of patients in both inpatient and outpatient department (*p* < 0.005). Maximum number of cases was found in the children of age group 1 to 4 years (Table 1). *Escherichia coli* (*n* =739, 68.5%) was the most common organism isolated from urinary tract infections in pediatric group in this study.

**Table 1:** Pediatric patients with Urinary tract infections (*N* = 1079)

Patients with Urinary tract infections							
Age Group	Male (%)	Female (%)	*p*	Outpatient (%)	Inpatient (%)	*p*	*Cc*
<1	87	129	0.352	54	162	0.001	0.014
1 to 4	129	141	0.001	119	151	0.004	0.104
5 to 9	118	238	0.004	248	108	0.007	0.083
10 to 14	86	151	0.193	151	86	0.001	0.029
Total	420	659		572	507		

*Cc* Contingency coefficient

### Antimicrobial resistance pattern of *E. coli*

High level of drug resistance was noted in *E. coli* isolates. Among 739 *E. coli* isolated, highest resistance (87% each) were to ampicillin and cephalexin, followed by ciprofloxacin (78%), cefixime (71%) and levofloxacin (67%) respectively. Very few isolates (5%) were resistant to imipenem whereas entire strains revealed high susceptibility (100% each) towards colistin and tigecycline (Table 2).

**Table 2:** Antibiotic susceptibility of MDR, XDR and ESBL *E coli* isolates

	No. of resistant isolates (%)			
Antibiotics	Total isolates (%)	MDR (*n* = 480)%	XDR (*n* = 37)%	ESBL (*n* = 288)%
Ampicillin	645(87)	480(100)	37(100)	288(100)
Amoxicillin-clavulanate	355(48)	407(84.7)	37(100)	288(100)
Piperacillin-tazobactam	244(33)	91(19)	37(100)	78(27)
Cephalexin	645(87)	392(81.6)	37(100)	265(92)
Cefixime	525(71)	312(65)	37(100)	288(100)
Ceftazidime	333(45)	306(64)	37(100)	288(100)
Ceftriaxone	333(45)	306(64)	37(100)	288(100)
Aztreonam	318(43)	294(61)	37(100)	288(100)
Imipenem	37(5)	37(8)	37(100)	28(10)
Gentamycin	244(33)	130(27)	37(100)	118(41)
Amikacin	103(14)	64(13)	37(100)	52(18)
Ciprofloxacin	576(78)	387(80.6)	37(100)	225(78)
Levofloxacin	495(67)	245(51)	37(100)	155(54)
Cotrimoxazole	310(42)	158(33)	37(100)	141(49)
Tigecycline	0(0)	0(0)	0(0)	0(0)
Colistin	0(0)	0(0)	0(0)	0(0)

### Multidrug resistant (MDR) and Extensive drug resistant (XDR) isolates

Among total 739 *E. coli* isolates subjected for antimicrobial susceptibility testing, 480 (64.9%) isolates were found multidrug resistant (MDR) and 37 (5.0%) isolates were extensive drug resistant (XDR). MDR isolates were resistant to ampicillin (100%), amoxicillin clavulanate (84.7%), cephalexin (81.6%) and ciprofloxacin (80.6%) respectively. However, MDR isolates were susceptible towards amikacin (87%), imipenem (92%) and piperacillin tazobactam (81%). Although the number of XDR isolates was low, they were completely resistant to all antibiotics except colistin and tigecycline (Table 2).

### ESBL *E coli* and their susceptibility pattern

Extended spectrum beta lactamase (ESBL) enzyme was detected in 288(38.9%) *E. coli* isolates. Penicillins, cephalosporins and monobactam group of antibiotics were appeared completely ineffective (100% resistance) against ESBL producers. However, ESBL producing *E coli* strains were susceptible to reserve class of antibiotics including imipenem (90%), colistin (100%) and tigecycline (100%) (Table 2).

## Discussion

To the best of our knowledge, this report represents the first description of ESBL producing uropathogenic *E. coli* involved in pediatric cases of urinary tract infections from our country, Nepal. Urinary tract infections are the most common infections in children and *E. coli* being leading pathogenic agent in these infections; it was our matter of interest. There was no previous report before this study to estimate the most common pathogen and its resistant pattern in pediatrics patients with urinary tract infection in our hospital.

The incidence of urinary tract infection based on significant bacterial growth among pediatric patients in this study was 19.6% and *E. coli* (68.5%) was the predominant pathogen. Similar rates have been previously reported from nearby hospitals [11, 12] and from studies of other countries [18–22]. Concurrently, significantly more females (61.0%) were found with UTI corroborating with other similar studies [12, 19, 20]. In our study, children of age group 1-4 years were found with highest number of UTI cases (contingency coefficient 0.104). Similar study from nearby hospital also reported that children less than six years of age were found UTI prone [11]. Urinary tract infection was significantly more prevalent in the female children of age group 1-4 and 5-9 years and also, more inpatients were found with UTI (*p* < 0.05). The higher rates of UTI in this age group might be due to immune status, sanitation, and ascending infection with fecal flora.

The high prevalence of ESBL-producing uropathogenic *E. coli* (38.9%) among children is reported in this study. In addition, this study also documents the enhanced resistance of ESBL producing *E. coli* to other antimicrobial groups like aminoglycosides and fluoroquinolones. Indeed, variations in the prevalence rates of ESBL-producing *E. coli* isolates in children around the globe and even among different hospitals within a country have been reported. Our prevalence rate of ESBL producing *E coli* (38.9%) is close to the findings reported by other studies in different parts of Asian region including Shettigar et al. (37.7%) from India [22], Pourakbari et al. (37%) and Rezai et al. (30.5%) from Iran [21, 23], Moore et al. (44%) from Cambodia [19] and Kizilca et al. (41.4%) from Turkey [24]. Extremely higher rates of ESBL *E coli* have also been reported, notably by Chinnasami et al. (83%) from India [25], Masud et al. (53.8%) from Bangladesh [20] and Shah et al. (50.9%) from Pakistan [18]. The increased rate of ESBL-producing bacteria causing infection in community as well as hospital settings constitutes an undeniable trend. Worldwide, pediatric UTIs due to ESBL-producing bacteria are an important part of this problem because they limit therapeutic choices and increases morbidity of infection [26]. However, lower rates of ESBL-producing *E. coli* were also reported, particularly from developed countries including 9.3% from USA [27], 10.2% from Korea [28], 14% from Taiwan [26], 14.1% from Lebanon [5] and 20.2% from Turkey [29]. These variations in the rate of ESBL producing strains of *E coli* among UTI cases might be attributable to the geographical difference, local antibiotic prescribing policy, the extensive use of broad spectrum antibiotics especially third generation cephalosporins and endemicity of drug resistance pathogens in the locality.

ESBL producing bacteria causing infections in children may have various complications and adverse outcomes [30]. ESBL producers are non susceptible to aminopenicillins and ureidopenicillins as well as extended-spectrum β-lactam agents like second- and third-generation cephalosporins. Use of these agents as the first choice for the treatment of urinary tract infections may lead to the inappropriate treatment and predispose to long term renal complications [24]. Therefore, antimicrobial therapy in infections with ESBL producing organism is really challenging. Published reports showed that ESBL- producing strains causing UTI in children associated with prior hospitalization, beta-lactam therapy, catheterization, underlying co-morbidity and infancy [24].

In this study, multidrug resistant (MDR) and extensively drug resistant *E coli* were found 64.9% and 5.0% respectively. Increasing pattern of resistance of urinary tract pathogens against common antibiotics in Nepal have also been reported by other researchers [12, 31] but MDR rates and drug resistance pattern among pediatric isolates from Nepal was not available. It is observed that ampicillin, cephalexin, ciprofloxacin and cefixime were poorly effective against uropathogenic *E coli*. Only 13% of the isolates were found susceptible to all the antibiotics tested. Cephalosporin, the commonly prescribed antibiotic as empirical therapy in pediatric and adults, resistance to this group of antibiotics was found high. Almost 45% of *E coli* isolates were resistant to at least one cephalosporin and monobactam. Similar rates of antimicrobial resistance was documented in the study from Bangladesh [20], Iran [32] and India [14]. However, compared to previous reports from Nepal, we observed a considerable increase in resistance against penicillins, aminoglycosides, quinolones and ceftriaxone [12, 31]. Lower rates of resistance among the pediatric isolates causing UTI have been documented in western countries [33].

Higher resistance to penicillins third generation cephalosporins in this study has been attributable to ESBL production among gram negative isolates. In ESBL producing isolates, augumentins (combined with beta lactamase inhibitor) such as amoxicillin clavulanate or piperacillin tazobactam can be used as alternative antimicrobials [34]. However, in this study, alarming state of resistance was observed among ESBL producers towards amoxicillin clavulanate (100%) and piperacillin tazobactam (27%). In the case when UTI is caused by an ESBL producing bacteria in children, the broadest-spectrum antibiotic agents such as carbapenems are recommended [35] but they are only useful in hospitalized patients. In this study, too, carbapenems were found effective to the ESBL isolates. Nevertheless, for pediatric UTIs in our setting, cotrimoxazole, amoxicillin clavulanate, ciprofloxacin and amikacin can still be used as first line therapy. Furthermore, other non carbapenem groups of antibiotics in UTIs due to ESBL-producing strains have also been described [36, 37]. ESBL stable cephamycins, fosfomycin and nitrofurantoin were shown effective for UTIs caused by ESBL-producing strains but their clinical utility as monotherapy is controversial [38–40]. In addition, ESBLs usually confer resistance to other classes of antibiotics, such as quinolones and trimethoprim/sulfamethoxazole, therefore susceptibility testing of these agents is important [23]. In this study, entire MDR isolates were resistant to ampicillin and 33% isolates were resistant to cotrimoxazole, 19% to piperacillin tazobactam and 8% to imipenem whereas no isolates were found to be resistant to colistin and tigecycline. Similarly, all XDR isolates were resistant to most of the antimicrobials tested whereas colistin and tigecycline were the most effective regimens against XDR isolates. Similar rate of resistance has been documented by Ansari et al. [41] but their study included *E coli* isolates from all age groups.

The level of drug resistance in uropathogenic *E coli* among pediatric patient in this study is a serious issue. Previous reports have suggested that higher resistance is likely to be occurring in the communities with higher proportion of young children and high antibiotic consumption [42]. In Nepal, higher antimicrobial pressure for community infections and inappropriate therapeutic guidelines for pediatric patients might be attributable to this menacing scenario [12, 31]. Resistance to the broad spectrum cephalosporins, fluoroquinolones and aminoglycosides among the ESBL producing *E.coli* isolates in this study necessitates the use of carbapenem as alternative choice for pediatric UTIs. Although we found carbapenems as the most effective agent against the ESBL but the high rate of resistance from similar studies is of special concern [41]. Furthermore, the genes associated with antibiotic resistance usually reside in plasmid and may transfer antibiotic resistance to other wild strains of bacteria [20]. Therefore, evidence based therapy with broad spectrum antibiotics for serious or critical cases to prevent bacterial resistance is extremely needful. Aminoglycosides, amoxicillin clavulanate and trimethoprim sulfamethoxazole/cotrimoxazole would be useful alternatives as empirical antibiotics for children suspected with UTIs in our scenario.

### Limitations of the study

This study has a number of limitations. We could not evaluate the risk factors and outcome of pediatric UTI cases in our setting. Further cohort studies with antimicrobial therapy and outcome would generate more significant results. Antimicrobial susceptibility testing by dilution methods and determination of minimum inhibitory concentration (MIC) of therapeutic antibiotics would be helpful for treatment and monitoring of the drug resistant infections. Due to unavailability of resources, we could not detect the genotype of ESBLs among *E coli* isolates. Further investigations with larger patient population and multiple centers would generate more significant ideas.

## Conclusion

We found the menacing state of drug resistance in almost all of the *E coli* isolates included in this study. Childhood UTIs caused by ESBL-producing *E. coli* has been emerged as a serious problem in our setting. Aminoglycosides and carbapenems can be used as alternative regimens for serious infections caused by MDR *E coli*. Furthermore, it is extremely necessary to formulate a strict antibiotics prescription policy and prudent use antibiotics in our country.

## Abbreviation

ASM: American Society for Microbiology
ATCC: American Type Culture Collection
CDT: Combined Disk Test
CLSI: Clinical and Laboratory Standard Institute
*E coli*: *Escherichia coli*
ESBL: Extended spectrum beta-lactamases
MDR: Multidrug resistant
MHA: Mueller Hinton Agar
MIC: Minimum inhibitory concentration
UTI: Urinary tract infection
XDR: Extensive drug resistant
